# Scapular renal cell carcinoma metastasis as a cause of high-output heart failure: a case report

**DOI:** 10.1186/s12872-022-02588-8

**Published:** 2022-04-05

**Authors:** Rémy Hamdan, Vincent Petit, Sylvie Zanetta, Jean-Christophe Eicher, Mathieu Mourot

**Affiliations:** 1 Department of Angiology, Dijon Bourgogne University Hospital, 14, Rue Paul Gaffarel, 21000 Dijon, France; 2 Department of Oncology, Georges-François Leclerc Cancer Centre, 1 Rue Professeur Marion, BP 77 980, 21079 Dijon, France; 3 Department of Cardiology, Dijon Bourgogne University Hospital, 14, Rue Paul Gaffarel, 21000 Dijon, France

**Keywords:** High-output heart failure, Metastatic renal cell carcinoma, Arteriovenous shunts, Hemodynamic, Doppler ultrasound, Case report

## Abstract

**Background:**

High-output heart failure is a rare condition that occurs when the heart is unable to respond to a sustained increase in blood demand. On echocardiography, a cardiac index of > 4 L/min/m^2^ (or 6 L/min) is a clear indicator of this disorder. The causes of high-output heart failure vary, but they all involve peripheral vasodilation or arteriovenous shunting. Renal cell carcinoma is well known for producing high levels of angiogenic growth factors that induce arteriovenous shunts. The decrease in peripheral arterial resistance and the increase in venous return result in a permanent high cardiac output, followed by congestive heart failure. Single bone metastases of renal clear cell carcinoma tumours causing high cardiac output and heart failure symptoms have been reported less than ten times in the medical literature.

**Case presentation:**

Before a right-shoulder painful lump with a murmur when auscultated, magnetic resonance imaging revealed a large scapular mass, which was biopsied and found to be a bone metastasis of renal cell carcinoma. Two months later, the patient developed heart failure for the first time. There was no evidence of cardiac disease on echocardiography. The cardiac output was 9.8 L/min and the cardiac index was 5.1 L/min/m^2^. Doppler ultrasound revealed numerous arteriovenous shunts in the large scapular metastasis and a right axillary artery flow of 24% of cardiac output. Sustained lower cardiac output was obtained following lesion-focused radiotherapy and systemic antiangiogenic treatment with axitinib and pembrolizumab.

**Conclusions:**

Herein, we present a unique case of high-output heart failure in a 70-year-old man diagnosed by echocardiography and upper-limb Doppler ultrasound in the context of metastatic renal cell carcinoma without pre-existing cardiac disease. We stress the potentially life-threatening hemodynamic consequences of hypervascularity associated with arteriovenous shunts within a single metastatic renal cell carcinoma implant, the importance of auscultating any progressing bone mass, and the utility of non-invasive Doppler ultrasound assessment in this setting.

**Supplementary Information:**

The online version contains supplementary material available at 10.1186/s12872-022-02588-8.

## Background

Chronic heart failure (HF) is a condition in which the heart is unable to satisfy the body’s demand for blood. The most common form, categorized as HF with reduced ejection fraction or HF with preserved ejection fraction, is heart disease resulting in low cardiac output while blood demand remains normal. On the contrary, high-output HF occurs when the heart is unable to respond to a sustained increase in blood demand, irrespective of pre-existing heart disease. After a period of compensation by increasing heart rate and ejection volume, clinical signs of cardiac overload may appear. It is a relatively rare disorder, accounting for less than 1% of patients presenting clinical symptoms of HF [1, 2]. No specific therapeutic recommendations exist except for diuretics, and the treatment of the underlying condition generally takes precedence. The diagnosis should be suspected anytime the cardiac index is > 4 L/min/m^2^ on echocardiography (TTE) [1]. While a wide range of circumstances can result in high-output HF, the pathologic mechanism always implies peripheral vasodilation (obesity, thyrotoxicosis, chronic anaemia, chronic obstructive pulmonary disease) or arterio-venous shunting (acquired arteriovenous fistula, arteriovenous malformations, multiple myeloma) [1, 2]. Since 1953, there have been about forty reports of congestive HF caused by intra-tumour vascular shunts in the context of renal primitive cancer [3, 4]. In renal cell carcinoma (RCC), the von Hippel-Lindau tumor suppressor gene is silenced, causing hypoxia-inducible transcription factors to activate and the production of proangiogenic growth factors like vascular endothelial growth factor [5, 6]. As a result, vascular chambers can form inside the tumor, connecting arteries and veins and causing blood shunts from arterial to venous circulation. Metastatic implants of RCC also contain shunts [6]. Multitargeted tyrosine kinase inhibitors (TKI), designed to inhibit vascular endothelial growth factor receptor, have been developed as a systemic treatment for metastatic renal cell carcinoma (mRCC). Yet, only six cases of patients presenting symptoms of HF imputable to shunts in a single bone metastasis of RCC have been reported [7–12]. Here we present an episode of HF in a 70-year-old man due to massive arteriovenous shunts in a large scapula metastasis two months after the diagnosis of mRCC. Clinical examination, echocardiographic data, and Doppler ultrasound (US) evaluation of the upper limb vessels and the right scapular mass were used to establish the diagnosis. Written informed consent was obtained from the patient before this text was prepared.

## Case presentation

**Fig. 1:**
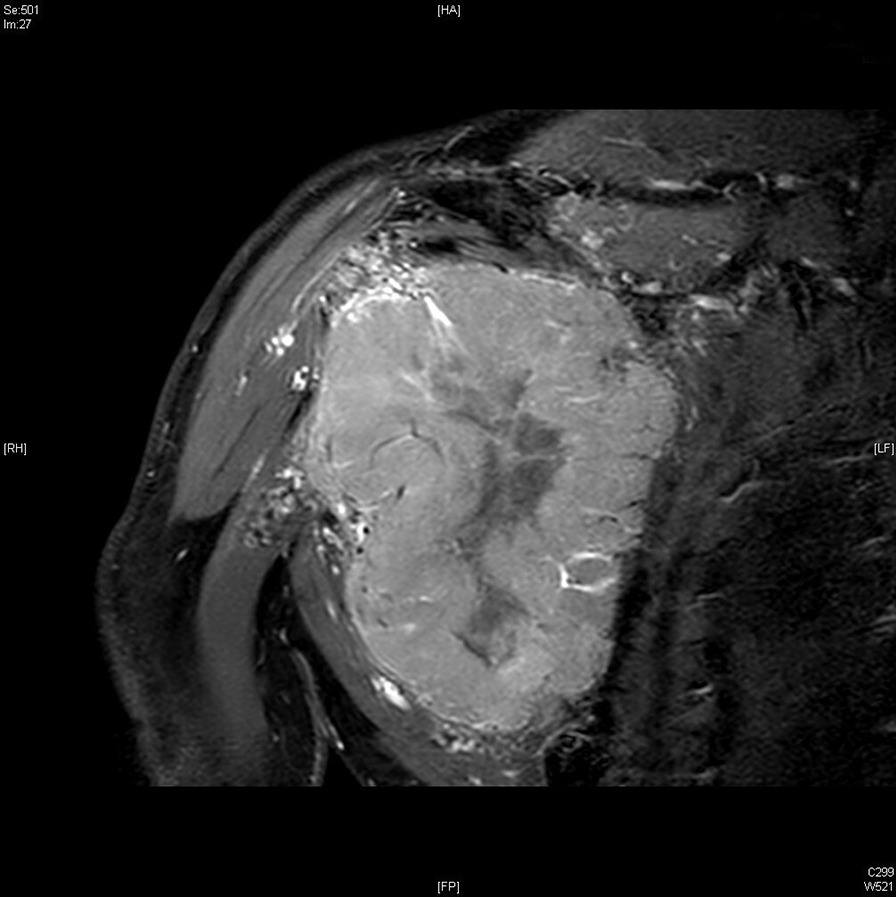
Magnetic resonance imaging of the right scapular mass

**Fig. 2:**
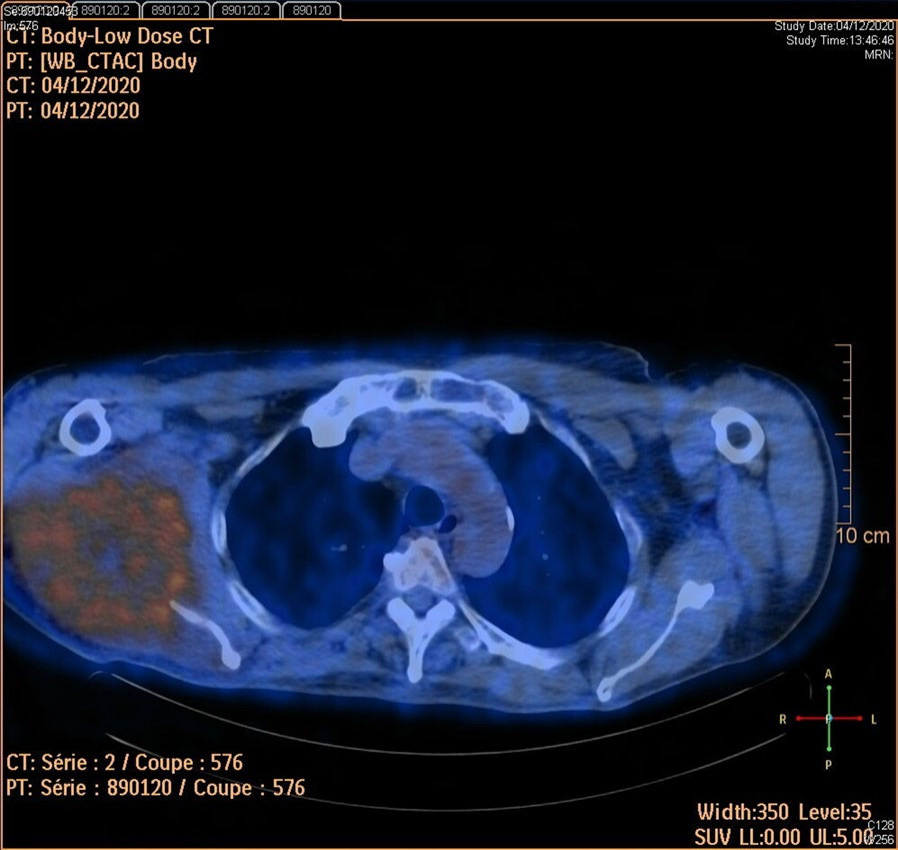
Positron emission tomography images of the right scapular mass

**Fig. 3:**
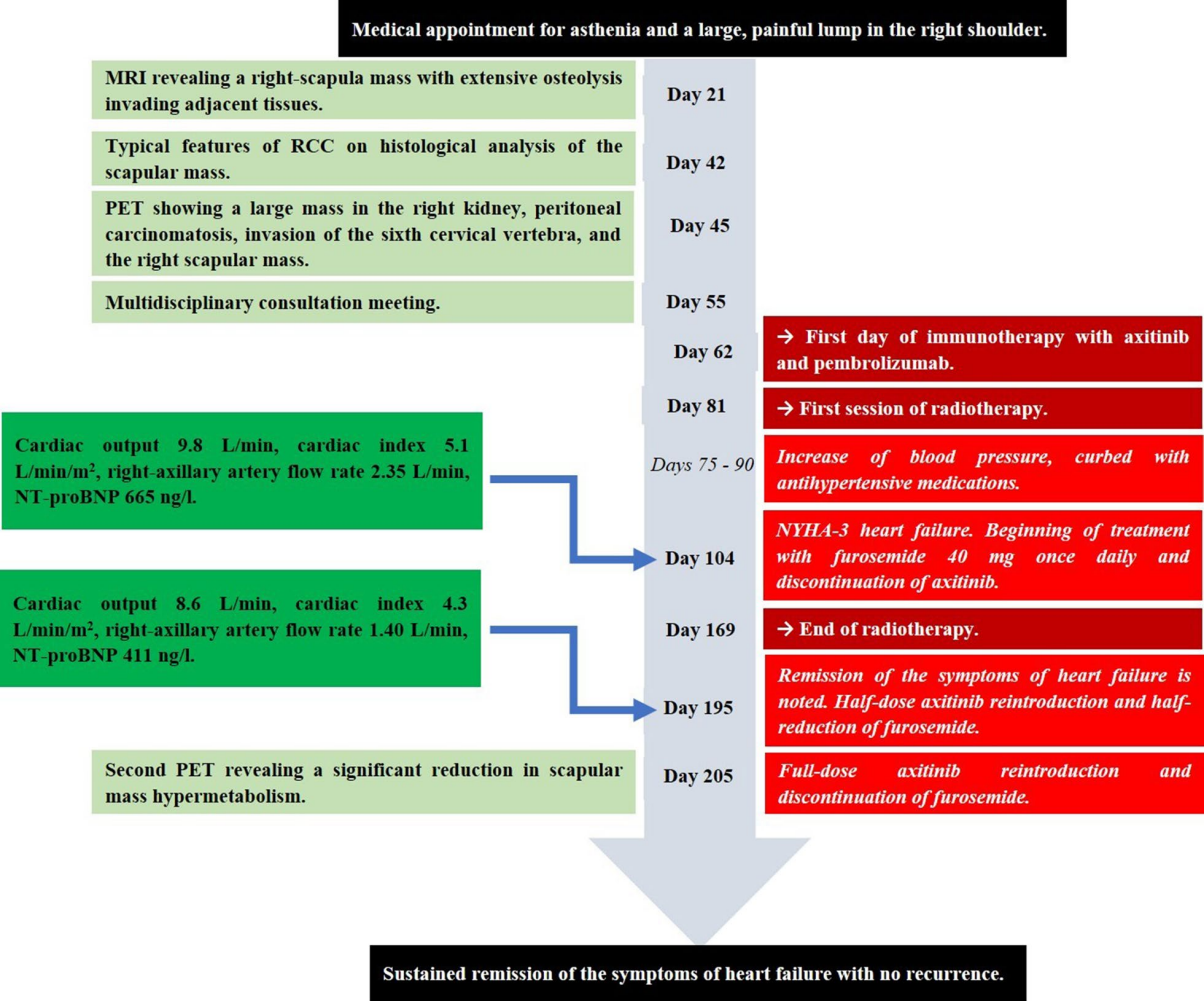
Information from this case report organized into a timeline figure

**Fig. 4:**
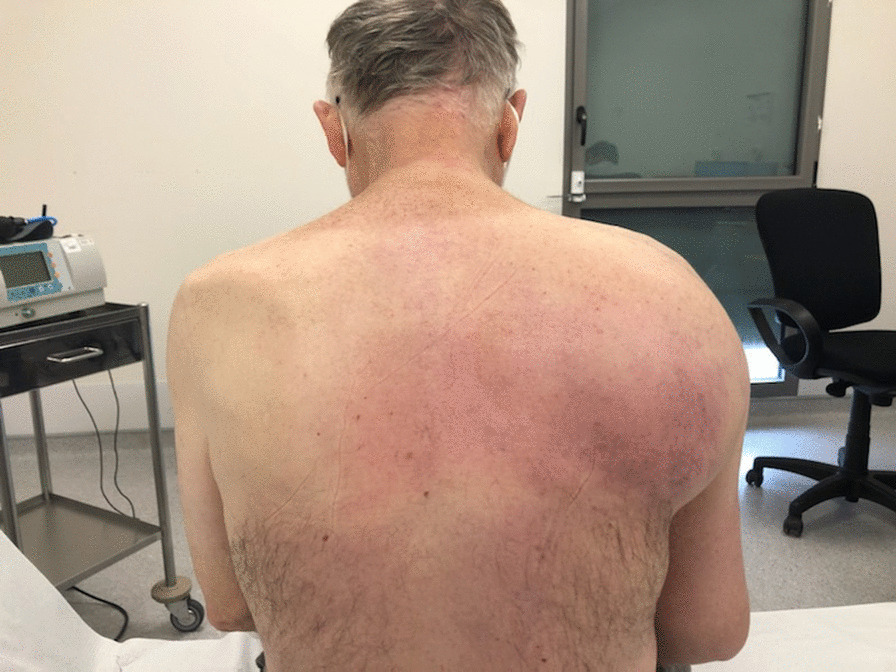
Clinical aspect of the right scapular mass

**Fig. 5:**
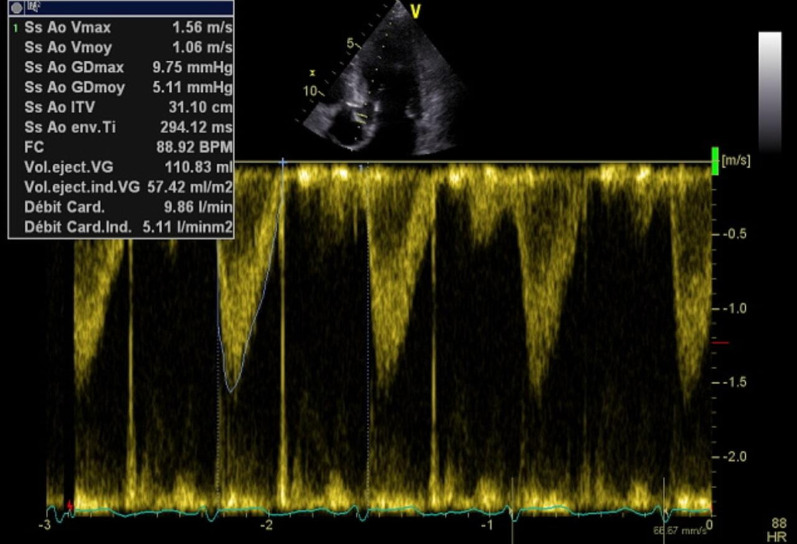
Transthoracic echocardiogram image: cardiac output measured at 9.8 L/min in two-dimensional imaging (stroke volume multiplied by heart rate)

**Fig. 6:**
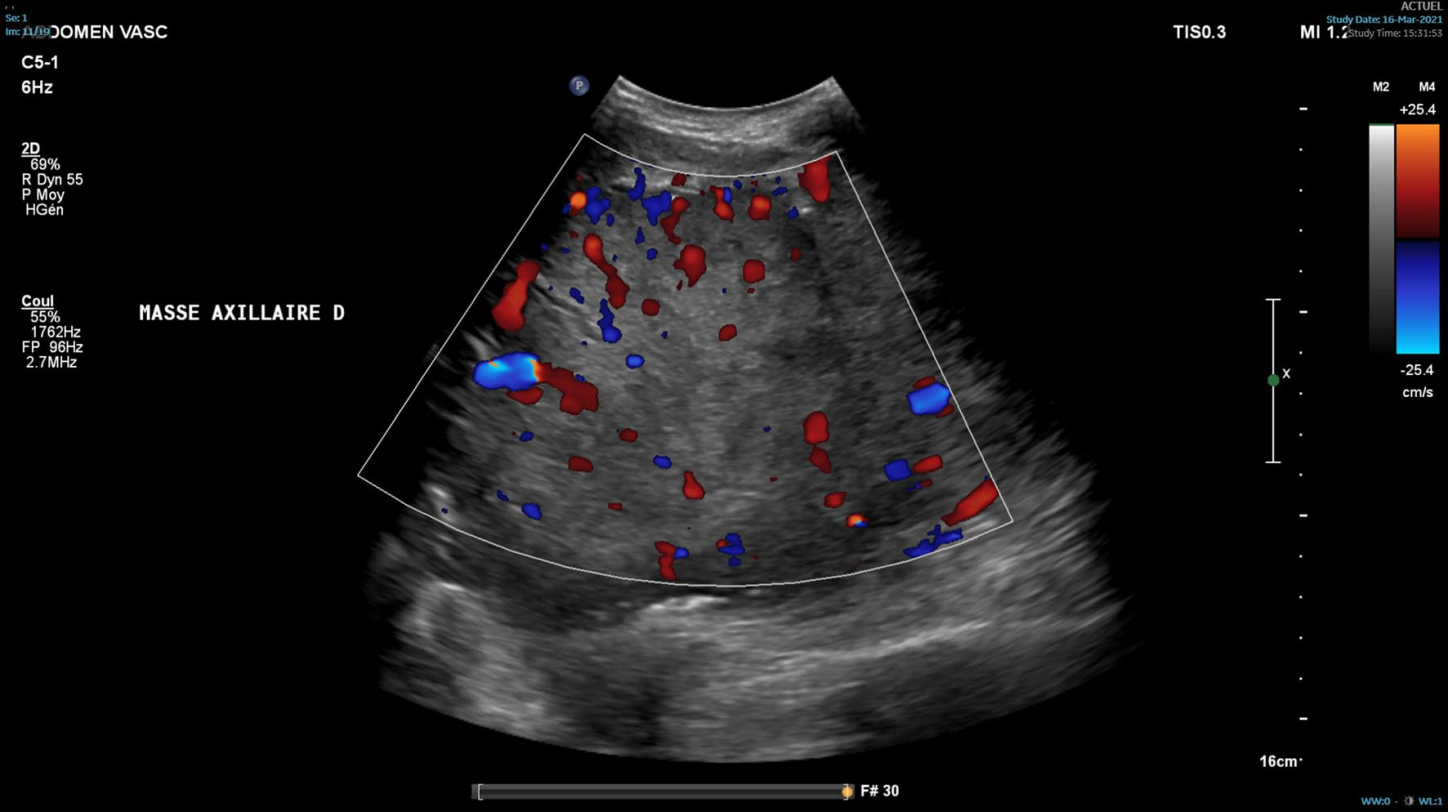
Doppler ultrasound image of numerous arteriovenous shunts within the right scapular mass

**Fig. 7:**
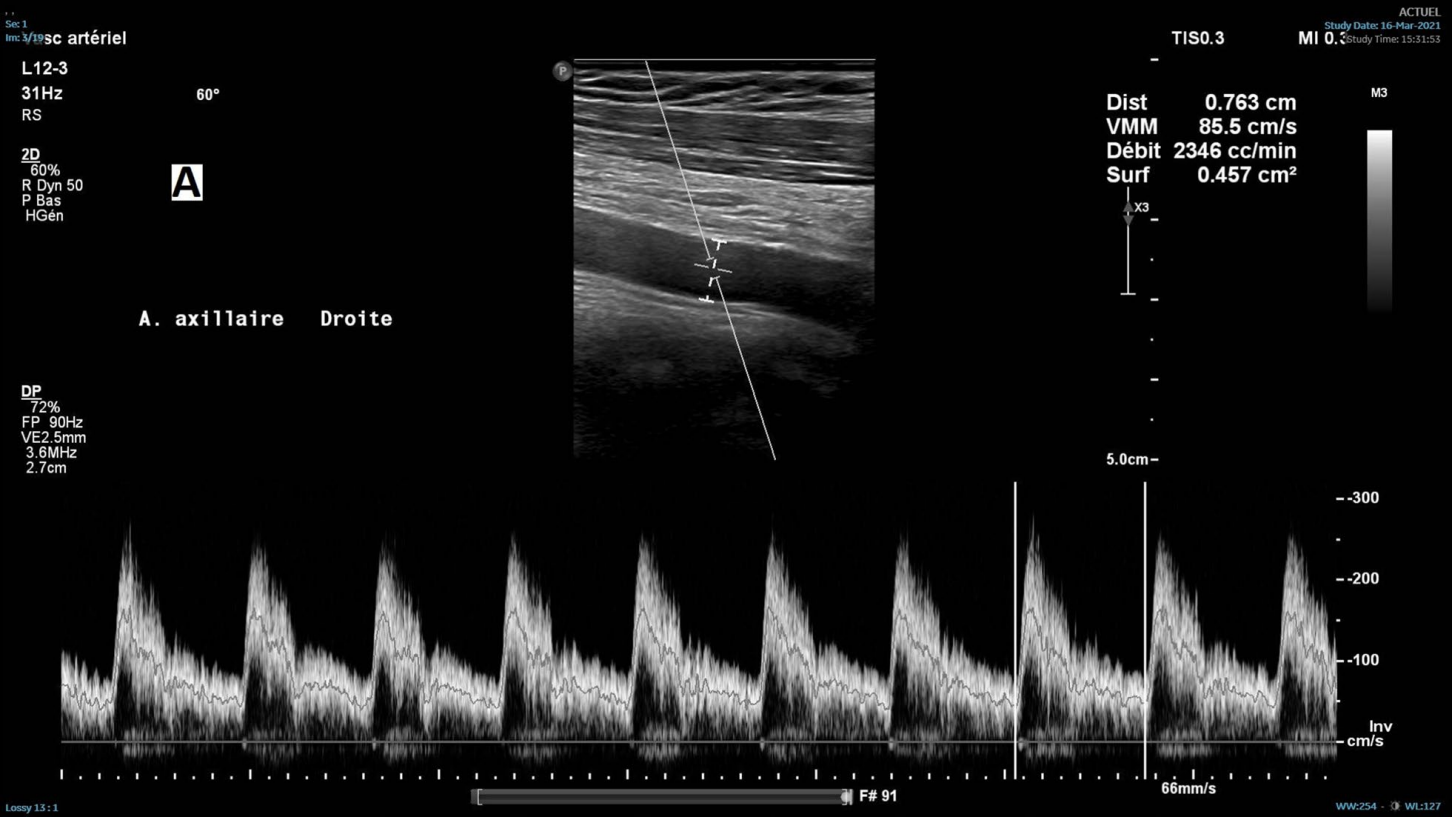
Systolo-diastolic Doppler velocity waveform in the right axillary artery with estimated flow of 2.35 L/min

**Fig. 8:**
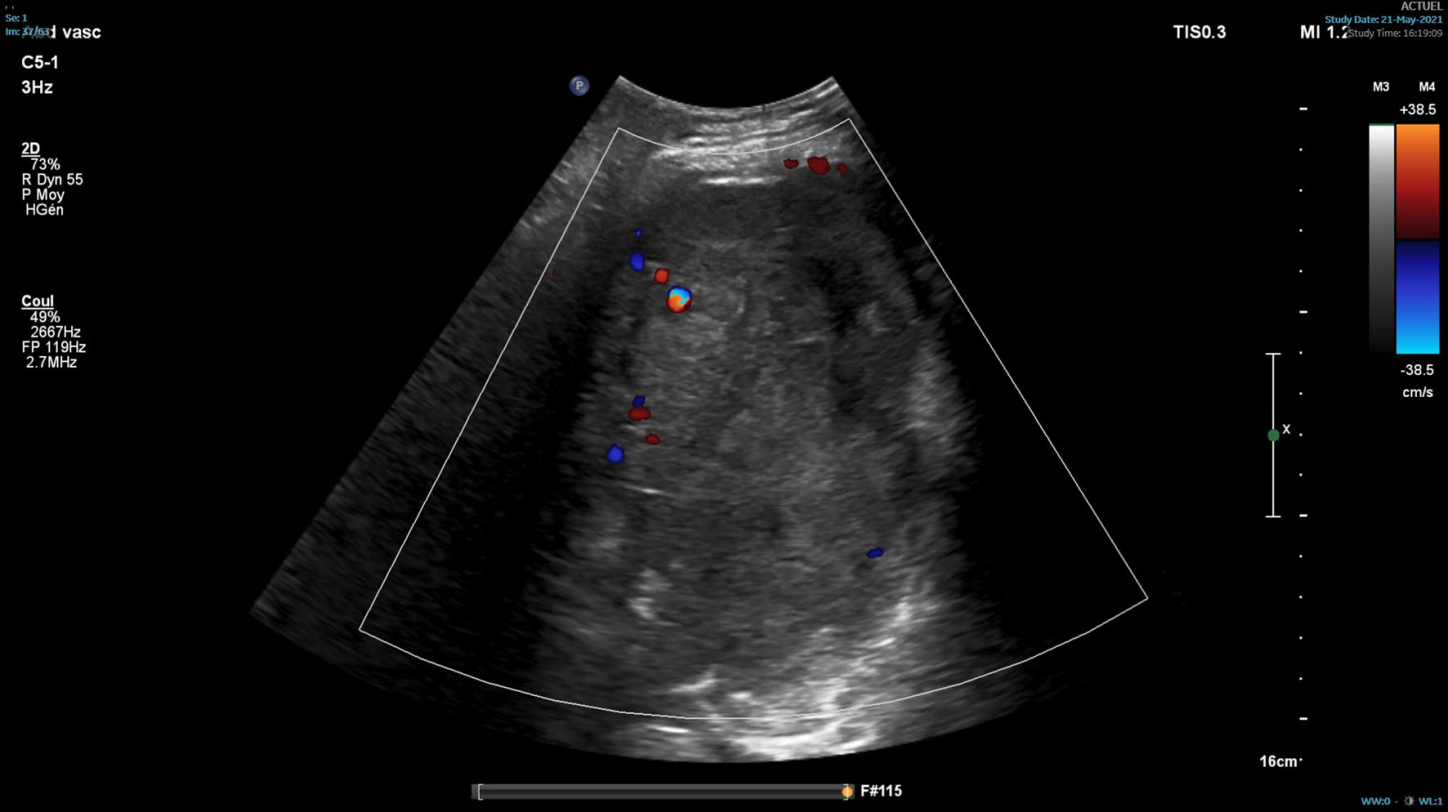
Doppler ultrasound image of the right scapular mass after thirteen sessions of lesion-focused radiotherapy showing less intra-metastatic arteriovenous shunts

The patient consulted his general practitioner about a large, painful lump, loss of motor function in the right shoulder, and asthenia. Auscultation of the mass revealed a continuous murmur. The medical history included hypertension, type-2 diabetes, and a transient ischemic attack four years previously. A thirteen-centimetre tissue lesion with extensive osteolysis of the right scapula invading the soft tissue and the glenohumeral capsule was discovered using magnetic resonance imaging (MRI) (Fig. 1). Two microbiopsies of the scapular mass revealed a tumour proliferation of medium-sized, quadrangular cells with clear cytoplasm, typical of RCC. Positron emission tomography (PET) unveiled a large mass (measuring 15*9*12 cm) involving the antero-inferior lobe of the right kidney, nodules of peritoneal carcinomatosis in the left paracolic gutter and near the spleen, a mass invading the left lateral pedicle of the sixth cervical vertebra, and the right scapular mass invading the shoulder girdle (Fig. 2). In line with the current recommendations [13], the multidisciplinary consultation meeting validated a therapeutic strategy including radiotherapy focused on the right scapula and the spinal cord (twenty gray in thirteen sessions) as well as systemic treatment with axitinib plus pembrolizumab. After initial destabilization, we were able to equilibrate the patient's blood pressure (Fig. 3). However, six weeks later, the patient developed ankle swelling and fatigue while gaining ten kilograms within a few days. Ordinary activities were resulting in undue breathlessness, limiting the activities of daily life (class III of the New York Heart Association's functional classification). Self-blood pressure monitoring found daily systolic blood pressures between 130 and 140 mmHg. Pulmonary crackles were bilaterally perceived on pulmonary auscultation. The right scapular mass was still prominent and very painful (Fig. 4). Blood and urinary tests showed normal serum albumin, normal creatinine levels, normal liver function tests, and no proteinuria. N-terminal pro-B-type natriuretic peptides (NT-proBNP) were 665 ng/l and troponins were 0.07 μg/L (normal: less than 0.6 μg/L). The electrocardiogram (ECG) was normal, with a heart rate of 91 bpm and a normal cardiac rhythm. A transthoracic echocardiogram (TTE) depicted a non-dilated, non-hypertrophic left ventricle with excellent overall and segmental kinetics (Additional file 1: Video 1), an estimated ejection fraction of 60%, a dilated left atrium of 24 cm^2^, elevated left ventricular filling pressures, a dilated and non-compliant inferior vena cava, and an estimated systolic pulmonary artery pressure of 40–50 mmHg. The cardiac output was 9.8 L/min (normal range 4–6 L/min) and the cardiac index was 5.1 L/min/m^2^ (normal range 2.6–4 L/min/m^2^) (Fig. 5). These findings were consistent with a state of high-output HF. Doppler US examination of the right scapular mass showed a well-delineated mass with a 14-cm long axis, containing numerous arteriovenous shunts predominantly in the upper-region of the lesion (Fig. 6). Spectral Doppler analysis of the arteries of the upper limbs was highly asymmetrical, given the systolic diastolic run-off and elevated velocities in the subclavian artery and the axillar artery of the right upper limb. Furthermore, widened dorsal scapular and circumflex scapular arteries were seen at the axillobrachial junction, and the velocities within their lumen were very high. The Doppler velocity waveform was physiological in the downstream brachial artery and symmetrical to the left brachial artery. Right axillary artery flow was measured at 2.35 L/min at the middle of its course (Fig. 7), while proximal brachial artery flow was normal at 51 ml/min, suggesting a blood flow shunt of 2.3 L/min between the right axillary artery and the downstream brachial artery. Also, there was no arterialized flow in the right axillary vein, inferior vena cava, or right renal vein. Considering that right axillary artery flow accounted for 24% of cardiac output, normal blood pressure, and the absence of any other identified cause, we attributed the condition of high-output HF-and subsequent cardiac overload-to the hypervascularity of the right scapular metastasis. Axitinib was discontinued due to its potential cardiovascular adverse effects, and furosemide was introduced at a dose of 40 mg per day (Fig. 3). Ongoing diuretic treatment successfully reduced breathlessness and peripheral oedema. The patient completed the thirteen planned radiotherapy sessions. A half-dose of axitinib was reintroduced three months after it was discontinued, and the dose of furosemide was reduced by half (Fig. 3). The patient reported feeling better, with no dyspnea, less pain, and improved motor function in the right arm. The pulmonary auscultation returned to normal. The NT-proBNP level decreased to 411 ng/L. Additional Doppler US examination highlighted a right-arm axillary outflow estimated at 1.40 L/min and a dramatically depressed diastolic component. The mass was still 14 cm in diameter, but the arteriovenous shunts were reduced (Fig. 8). A second TTE showed normal left ventricular filling pressure with a measured cardiac output of 8.6 L/min and a cardiac index of 4.3 L/min/m^2^. The second PET revealed a significant reduction in right scapular hypermetabolism. Fifteen days later, the furosemide treatment was completely stopped. The symptoms of HF never recurred.

## Discussion

25% of patients with RCC have metastases when they are first diagnosed [14]. Bone metastases are the second most common type [14], and the scapula is commonly involved [15]. In the few reported cases of high-output HF due to a single bone metastasis of RCC, the diagnosis was based on pathological analysis of a resection specimen [8], direct visualisation of arteriovenous fistulas by angiogram [10, 12], measurement of intracavitary pressures by direct cardiac catheterization [12], or through autopsy [7]. The treatment was always for the cause, consisting of resection of the mass [10, 11] or radiotherapy [9, 12]. In our case, interestingly, while cardiac output dropped by only 1.2 L after treatment, remaining above the physiological norm, the NT-proBNP level returned to normal, and HF symptoms disappeared. In our opinion, immunotherapy's antiangiogenic effects, combined with radiotherapy, reduced scapular hypervascularization, lowering blood demand in the right upper limb and, mechanically, the cardiac output. As a result, the patient was able to return to a subclinical state of high cardiac output. TKI therapies emerged about twenty years ago and are now widely used in the treatment of mRCC. Cardiovascular complications, such as hypertension and left ventricular dysfunction, have been reported [16]. Axitinib-induced cardiopathy is either treatment-induced hypertensive heart disease, ischaemic cardiopathy due to antiangiogenic effects on the coronary arteries, or immune-related myocardiopathy, with echocardiographic evidence of left ventricular dysfunction [17, 18]. In our report, clinical examination, MRI, and PET data, which were correlated with TTE and upper-limb Doppler US data, were used to make the diagnosis. Although the blood pressure was found to be uncontrolled for a few days two weeks after starting immunotherapy (grade II adverse effect), it rapidly ceased, self-measured blood pressure was strictly normal in the following weeks, and signs of HF appeared two weeks later (Fig. 3). During the episode of HF, both arms' blood pressures were normal, the ECG was unchanged, there was no chest pain, troponins were negative, and ejection fraction was preserved on TTE with no argument for a heart disease. As a result, we believe that the imputability of axitinib is negligible. This episode of HF is all the more due to the hypervascularity of the scapular mass because, concomitantly to the complete clinical remission, Doppler US confirmed a reduction of the intra-tumoral arteriovenous shunts, and a one-litre decrease in right axillary flow correlated with a one-litre decrease in cardiac output on TTE. Doppler US not only allowed the type and aetiology of HF to be determined, but it also allowed the effectiveness of treatment to be accurately and non-invasively monitored.

## Conclusion

Given the potentially life-threatening hemodynamic consequences of hypervascularity associated with arteriovenous shunts within a metastasis of RCC, any rapidly growing skeletal mass requires prompt auscultation, and if a murmur is heard, kidney cancer should be considered. In this report, we detail a rare case of high-output HF secondary to scapular metastasis of RCC and sustainably controlled with scapular lesion-focused radiotherapy and a combination of axitinib plus pembrolizumab. Doppler US was useful for diagnosis and assessing the treatment's effectiveness.

## Supplementary Information

**Additional file 1. **Apical four-chamber view on TTE: apical four-chamber view of systole recorded during the first transthoracic echocardiogram (day 104), showing excellent overall kinetics.

## Data Availability

The data used to write this article may be shared upon reasonable request to the corresponding author.

## Abbreviations

HF: Heart failure
RCC: Renal cell carcinoma
mRCC: Metastatic renal cell carcinoma
US: Ultrasound
MRI: Magnetic resonance imaging
PET: Positron emission tomography
NT-proBNP: N-terminal pro-B-type natriuretic peptide
ECG: Electrocardiogram
TTE: Transthoracic echocardiogram
TKI: Tyrosine kinase inhibitors

## References

[1] Mehta PA, Dubrey SW (2009). High output heart failure. QJM.

[2] Reddy YNV, Melenovsky V, Redfield MM, Nishimura RA, Borlaug BA (2016). High-output heart failure: a 15-year experience. J Am Coll Cardiol.

[3] Hayek S, Kung R, Barb I, Master V, Al S, Clements S (2014). Digging deep: high output heart failure in renal cell carcinoma. Am J Med.

[4] Tobe A, Tanaka A, Yoshida S, Kondo T, Morimoto R, Furusawa K, et al. High-output heart failure caused by a tumor-related arteriovenous fistula: a case report and literature review. Intern Med. 2021. 10.2169/internalmedicine.6962-20 PMC8502649 33776013

[5] Facchini G, Perri F, Caraglia M, Pisano C, Striano S, Marra L (2009). New treatment approaches in renal cell carcinoma. Anticancer Drugs.

[6] Howlett SA, Caranasos GJ (1970). Metastatic renal cell carcinoma producing arteriovenous shunt. Arch Intern Med.

[7] Lewis T (1940). A note on pulsating manubrial tumour. Heart.

[8] Fredell EW, Stone AO (1956). Pulsating lesions metastatic from renal cancer. Calif Med.

[9] Bourne G (1957). Increased cardiac output caused by secondary hypernephroma. Heart.

[10] Thomas CV, Feint JF, Nayman J (1965). An arteriovenous shunt associated with an adenocarcinoma of the kidney. Br J Surg.

[11] Vetto RM, Bigelow JC, Dueger WC (1966). Arteriovenous fistula in pulsatile metastatic carcinoma: report of chronic heart failure relieved by resection. Am Surg..

[12] Taboada CF, Garcia E, Pranke DW (1972). Metastatic hypernephroma and congestive heart failure. S Med J.

[13] Ljungberg B, Albiges L, Abu-Ghanem Y, Bensalah K, Dabestani S, Fernández-Pello S (2019). European Association of urology guidelines on renal cell carcinoma: the 2019 update. Eur Urol.

[14] Bianchi M, Sun M, Jeldres C, Shariat SF, Trinh Q-D, Briganti A (2012). Distribution of metastatic sites in renal cell carcinoma: a population-based analysis. Ann Oncol.

[15] Jacobsen KD, Follerás G, Fossá SD (1994). Metastases from renal cell carcinoma to the humerus or the shoulder girdle. Br J Urol.

[16] Qi W-X, He A-N, Shen Z, Yao Y (2013). Incidence and risk of hypertension with a novel multi-targeted kinase inhibitor axitinib in cancer patients: a systematic review and meta-analysis: axitinib associated hypertension. Br J Clin Pharmacol.

[17] Touyz RM, Herrmann J (2018). Cardiotoxicity with vascular endothelial growth factor inhibitor therapy. NPJ Precis Oncol.

[18] Tanriverdi O, Ates S, Sandal KK, Uylas S, Bosna IC, Alkan A (2020). Left ventricular dysfunction associated with axitinib and nivolumab experience in an advanced renal cell carcinoma. J Oncol Pharm Pract.

